# Neuroanatomical Correlates of Creativity: Evidence From Voxel-Based Morphometry

**DOI:** 10.3389/fpsyg.2019.00155

**Published:** 2019-02-04

**Authors:** Wenfu Li, Gongying Li, Bingyuan Ji, Qinglin Zhang, Jiang Qiu

**Affiliations:** ^1^School of Mental Health, Jining Medical University, Jining, China; ^2^School of Psychology, Southwest University, Chongqing, China

**Keywords:** creativity, remote associates test, voxel-based morphometry, anterior superior temporal gyrus, gray matter density

## Abstract

Creativity was a special cognitive capacity which was crucial to human survival and prosperity. Remote associates test (RAT), identifying the relationships among remote ideas, was one of the most frequently used methods of measuring creativity. However, the structural characteristics associated with RAT remains unclear. In the present study, the relationship between gray matter density (GMD)/white matter density (WMD) and RAT was explored using voxel-based morphometry (VBM) in a larger healthy college student sample (144 women and 117 men). Results showed that the score of RAT was significantly positively related with the GMD in the right anterior superior temporal gyrus (aSTG) and negatively correlated with the GMD in the right dorsal anterior cingulate cortex (dACC). Meanwhile, results also showed that the score of RAT was significantly positively related with the WMD in the right dACC and negatively correlated with the WMD in the left inferior frontal gyrus (IFG). These findings indicate that individual creativity, as measured by the RAT, was mainly related to the regional gray /white matter density of brain regions in the aSTG, dACC and IFG, which might have been involved in the forming of novel combinations, breaking of mental set, monitoring of conflict and semantic integration.

## Introduction

Creativity is an important cognitive ability which was crucial to human survival and prosperity (Ashtari and Cyckowski, 2012). It was considered as the creation of something unusual as well as potentially useful (Rex Eugene Sternberg and Lubart, 1993; Jung et al., 2013). The systematic exploration of creativity within psychology begins with the Guilford’s (Guilford, 1950) seminal address at the American Psychological Association (APA).

Creativity can be the result of divergent as well as convergent thinking (Guilford, 1950; Arden et al., 2010; Dietrich and Kanso, 2010). In the divergent thinking task, subjects were asked to generate multiple answers to open-ended questions, such as “describe what would happen if there is no sun” or “generate as many alternative uses as possible for brick”. In the convergent thinking task, participants were required to generate single answers to closed-ended problems, such as the Remote Associates Task (Mednick, 1962). The creative thinking process had been further defined “…as the forming of associative elements into new combinations which either meet specified requirements or are in some way useful” (Mednick, 1962). Mednick (1962) proposed the associative theory of the creative process and developed the remote association test (RAT), assessed the ability to identify relationships among remote ideas, as the form of a test of creative convergent thinking. Two university-level versions of RAT were constructed and each version consisted of thirty problems (Mednick and Mednick, 1967; Mednick, 1968). Each problem consisted of three stimulus words that can be combined with a fourth word in several ways, such as with synonymy, compound word and semantic association (Mednick and Mednick, 1967; Bowden and Jung-Beeman, 2003). Based on RAT, Bowden and Jung-Beeman (2003) had created a whole set of 144 Compound Remote Associate (CRA) problems which can be solved through formation of a compound word with all three given words. Overall, RAT or similar problems had been widely used in the investigation of insightful problem solving and creative thinking (e.g., Bowers et al., 1990; Schooler and Melcher, 1995; Kihlstrom et al., 1996; Ansburg, 2000; Qiu et al., 2008).

Previous studies indicate the importance of associative processing for convergent thinking (Mednick et al., 1964; Brown, 1973; Benedek et al., 2012). Higher associative fluency and more unusual association were found in more creative people rather than less creative people (Benedek et al., 2014). Other studies also found that there were more abundant and flexible connection networks in more creative people compared to less creative people (Carlsson et al., 2000; Jausovec and Jausovec, 2000). These inter-individual creative behavioral differences might be revealed by the structural brain imaging method (Kanai and Rees, 2011).

Recent investigations utilized electroencephalography (EEG) and functional magnetic resonance imaging (fMRI) to explore the neuromechanism of remote associates problems (for reviews see Arden et al., 2010; Dietrich and Kanso, 2010). Previous studies found that the superior temporal gyrus (STG) was consistently associated with creative thinking (Jung-Beeman et al., 2004; Kounios et al., 2008). For example, Jung-Beeman et al. (2004) investigated remote associate problems solving with fMRI and EEG. They found that increased brain activity in the right anterior STG was associated with insightful solutions relative to non-insightful solutions. With EEG they found that insightful problem solving was accompanied by high frequency EEG activity in the same brain region. These results might suggest that the STG area was involved in the linking of unrelated concepts together or the changing of representation.

A growing number of studies have centered on the inter-individual behavioral differences (Kanai and Rees, 2011) and its associated neuroanatomical correlates using non-invasive structural magnetic resonance imaging (sMRI) (e.g., Takeuchi et al., 2010a; Jung et al., 2010b; Li et al., 2015). T1-weighted imaging was the frequently- used sMRI sequence, which offered brain images with higher resolution and lower noise. Some indicators, regional gray matter density (rGMD), regional gray matter volume (rGMV) and cortical thickness, were obtained from T1-weighted image. Both rGMV and rGMD could be obtained from voxel-based morphometry (VBM) method which was used usually and possessed high validity in measuring brain structure. The rGMV measure reflected the absolute gray and white matter volume, whereas the rGMD reflected the relative gray and white matter concentrations (Takeuchi et al., 2011). The results of both rGMD and rGMV were typically similar (Good et al., 2001). These two kinds of measures were used frequently in brain anatomical researches (Andrea et al., 2005). Because of the gray matter which was thinning during natural maturing process (Sowell et al., 1999; Sowell et al., 2003; Sowell et al., 2014) and adolescents who were in the key period of cortical thinning (Ashtari and Cyckowski, 2012; Fuhrmann et al., 2015), rGMD was used more frequently than rGMV in studies in which subjects were adolescents.

Although creative thinking was a pervasive research topic in the domains of psychology and cognitive neuroscience, the neural basis of creativity remains largely unclear. Previous studies have investigated the neuroanatomical correlates underlying the measures of divergent thinking using sMRI. One review article indicated that convergent thinking was related to an increase as well as a decrease of cortical volume and thickness (Jung et al., 2013). Increased brain regions consisted of superior parietal lobule (Gansler et al., 2011), precuneus, midbrain, dorsolateral prefrontal cortex and striatum (Takeuchi et al., 2010a), and right angular gyrus and posterior cingulate (Jung et al., 2010b). Cousijn et al. (2014) further found that visuo-spatial divergent thinking was associated with increased cortical thickness in the right superior frontal gyrus and various occipital, parietal, and temporal areas. At the same time, Fink et al. (2014) explored the relationship between rGMD and divergent thinking and found that divergent thinking was positively correlated with the rGMD in the right cuneus and the right precuneus which might be involved in vivid imaginative ability in more creative individuals. Other studies further manifested that divergent thinking was associated with the increased rGMV in left and right inferior frontal gyrus (Zhu et al., 2013) and precuneus and caudate nucleus (Jauk et al., 2015) and the increased rGMD in the right precuneus and cuneus (Fink et al., 2014). These brain structure studies indicated that the structural basis of divergent thinking was associated with widely distributed brain regions (Dietrich, 2007; Takeuchi et al., 2017) and not a supporter of the notion of “more is better” (Jung et al., 2010a). These inconsistent results might be because of the complicacy of divergent thinking which depends on several cognitive functions (Dietrich, 2004; Dietrich and Kanso, 2010; Takeuchi et al., 2010b; Jung et al., 2013).

On the other hand, relatively few studies investigated the structural correlates of convergent thinking (e.g., RAT) using sMRI. Bendetowicz et al. (2017) employed 54 participants to investigate the brain anatomical basis of RAT and found the problem-solving associated with the decreased rGMV in the left rostrolateral prefrontal cortex as well as the left inferior parietal lobule. Ogawa et al. (2018) explored the neural basis of the insightful task (e.g., RAT) in a large sample (232 subjects) and found the task score correlated with the increased rGMV in the right insula and the middle cingulate cortex/precuneus and the decreased rGMV in the left crus 1 of cerebellum and the right supplementary motor area. A recent study explored the anatomical basis of remote association test in bipolar depression patients and found this test associated with the increased rGMV in the medial prefrontal gyrus. Although these results conflicted with previous fMRI study of convergent thinking (Jung-Beeman et al., 2004), it was consistent with the findings of divergent thinking associated with widely distributed brain regions. A recent meta analysis study investigated the neural basis of insight and found that extensive brain regions, containing the left inferior frontal gyrus and the amygdala, and the right medial frontal gyrus and the hippocampus, were activated by insight problem solving (Shen et al., 2018). Another review specialized in the function of the temporal lobe in insightful process and revealed an integrated-model on the role of different parts of temporal lobe in insight (Shen et al., 2017). However, no study investigating the association between convergent thinking and structural regions examined whether intelligence may also moderate the relationship between creative potential and brain structure in light of rGMD.

In the present study, we focused on the convergent thinking measured by the Chinese version of RAT modified from the compound association task (Bowden and Jung-Beeman, 2003) and the structural basis of convergent thinking using the measure of rGMD derived from VBM method. The latest study found that divergent thinking training could increase the rGMV in the dorsal anterior cingulate cortex (Sun et al., 2016). Other research indicated that frequently recruited brain regions would increase its volume (Maguire et al., 2000). We assumed that brain regions activated by convergent thinking would correspond with brain structural characteristic (e.g., increased and/or decreased rGMD) with the purpose of creativity performance. One of the goals of the present study was to confirm whether brain regions activated by compound remote association task in Mark Jung-Beeman et al. (2004) could be found again in the matter of rGMD. Previous studies indicated the importance of prefrontal gyrus for creativity. Besides, Broca’s area, a portion of inferior frontal gyrus, was known for involvement in language comprehension and production. Hence, the prefrontal gyrus or inferior frontal gyrus might likely to rediscovered. Consider that the anterior cingulate cortex was proved repeatedly and reliably by numerous studies to be involved in cognitive conflict detecting and mental set breaking (Dietrich and Kanso, 2010). Based on the notion that brain regions involved in some cognitive function would impact the efficiency and quality of the individual’s capacity to complete that function, the performance of RAT was assumed to be related with rGMD in anterior cingulate cortex which were certified to be crucial to insightful problem solving.

## Materials and Methods

### Participants

Two hundred and seventy-six university students (150 females and 126 males; the mean = 19.89 years, standard deviation = 1.28), who came from Southwest University (Chongqing, China), participated in the present research. The sample involved in our study was a part of Southwest University Longitudinal Imaging Multimodal (SLIM) data (for more details, please see: http://www.qiujlab.com), which was available for other investigators through the International Neuroimaging Data-sharing Initiative (INDI)^1^. The main purpose of this project was to explore the relationship among individual differences in brain structure and function, creativity, and mental health. The protocols of both behavioral and structural MRI were confirmed by the research ethics committee of Southwest University. The informed consent form was signed by participants before participating, which was authorized by the Institutional Human Participants Review Board of Brain Imaging Research Centre in Southwest University.

Nine subjects were removed because of the unfinished questionnaires of RAT and CRT. Another six participants were excluded because of the excessively large scanning artifacts and unnatural brain structures. Thus, 261 participants remained in the topological properties analysis. There were 117 males (mean age = 20.09 years, standard deviation = 1.33) and 144 females (mean age = 19.69 years, standard deviation = 1.23).

### Assessment of Convergent Thinking

The RAT was used to measure convergent thinking, which developed by Mednick (1962) as a means of measuring creativity considered with no need for knowledge specific to any field. We selected 25 items which were evaluated to be insightful (mean score > 1.8) on a scale of 1 (No-insightful feeling) to (strongest insightful feeling) for each items by another group of subjects (total 20). Each of the 25 items consists of three Chinese words that could be connected with an answer word in the way of formation of a compound word. For example, the three words “pai, mai, fan” (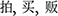) were connected with the solution word “mai” (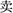) by way of the forming of compound word (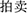, 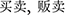). Reaching a solution needs creative thinking, because the information extracted from memory is usually wrong, and participants must come up with a more remotely related word for the purpose of problem solving. The intra-subject reliability was 0.719 measured by Cronbach’s alpha.

### Assessment of General Intelligence

The Combined Raven’s Matrices Test was used to test subject’s general intelligence and corrected for the possible effect of intelligence on brain structures (Haier et al., 2004). This test consisted of 72 items (Li, 1989). More details on what CRT were consisted and how CRT was performed could be found in our previous research (Li et al., 2016). The number of right answers, completed in 40 min, was regarded as the score of CRT.

### Data Acquisition

Siemens 3T scanner (MAGENTOM Trio, a Tim System) was used to scan subjects, which was located at the Brain Imaging Research Centre in Southwest University, Chongqing, China. Magnetization-prepared rapid gradient echo sequence was used to acquireT1-weighted structural MRI images (TR = 2530 ms, TE = 3.39 ms, TI = 1100 ms, flip angle = 7°, FOV = 256 × 256 mm, slice number = 128, in-plane resolution = 1 × 1 mm, slice thinkness = 1.33 mm).

### Imaging Data Preprocessing

VBM8 toolbox^2^ was used, and implemented in SPM8 software^3^ to perform the T1-weighted images. Firstly, each image was examined visually and six participants were removed on account of image quality (excessive scanner artifacts or gross anatomical abnormalities). Secondly, each subject image was adjusted manually to the anterior commissure (AC) and posterior commissure (PC). Thirdly, the “new segmentation” in SPM8 was used to segment the image into gray matter, white matter, cerebrospinal fluid and everything else (e.g., skull and scalp) followed the standard segmentation approach (Ashburner and Friston, 2005). Fourthly, the Diffeomorphic Anatomical Registration through Exponential Lie algebra (DARTEL) implemented in SPM8 was used to execute registration, normalization and smoothness analyses. The study-specific template was computed in registration analyses based on the average tissue probability maps. The images were then resampled to 1.5mm × 1.5 mm × 1.5 mm and normalized to the generated study-specific template which was in the MNI space. The normalized images were smoothed using an 8-mm full width at half maximum (FWHM) Gaussian kernel. The images, which represent the regional gray matter density and regional white matter density, were used for the following statistical analyses.

### Behavioral Data Analysis

The statistical software SPSS 13.0 (SPSS Inc., Chicago, IL, United Sates) was used to analyze behavioral data. The independent *t*-test was carried out to explore the gender differences in the score of RAT and CRT.

### MRI Data Analysis

The multiple regression analysis was used to investigate the relationship between convergent thinking and brain structure. The score of RAT was considered as the variable of interest and the gender, age and the score of CRT were entered simultaneously as the covariates as previous researches (R.E. Jung et al., 2010b; Fink et al., 2014; Li et al., 2015).

Multiple comparisons were calculated by using the Monte Carlo simulation-based Alphasim program (Cox, 1996; Ward, 2000), which was included in the REST toolbox^4^ (Song et al., 2011) and similar to the AlphaSim in AFNI. The threshold was set at *P* < 0.05 by combining the voxel-wise *P* < 0.005 and cluster size > 310 voxels (using the global gray matter mask, FWHM = 8 mm, cluster connection radius = 5 mm and 1000 iterations). Generally, AlphaSim was widely used in previous literatures about VBM data analysis (DeYoung et al., 2010; Schwartz et al., 2010; Ding et al., 2012; Zou et al., 2012; Farb et al., 2013; Kong et al., 2013; Yang et al., 2013). Although there might be some limitations with Monte Carlo simulation (Silver et al., 2011), it reduced the rate of false-positive results using the cluster-level threshold.

## Results

### Results of Behavioral Data

**Figure 1:**
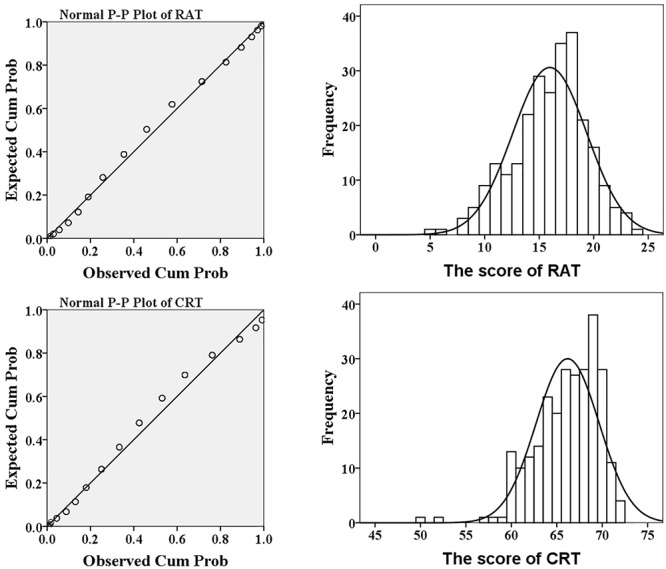
The P-P plot and histogram of the score of RAT (upper) and CRT (lower).

The results of descriptive analysis of age, the scores of RAT and CRT were displayed in Table 1. Two-sample *t* tests revealed that there were no gender differences in the score of RAT and the score of CRT (*Ps* > 0.1). The P-P plot and frequency histogram with a normal distribution curve of the score of RAT and CRT were shown in Figure 1. The Skewness of the scores of RAT and CRT were -0.414 and -0.490, respectively and the Kurtosis was 0.067 and -0.539, respectively. These results showed that both the scores of RAT and CRT were approximately normal distribution.

**Table 1:** Participant demographics (*N* = 261; men = 117, women = 144).

Measure	Mean	*SD*	Range


Age	19.86	1.29	17–27


RAT	15.97	3.40	5–24


CRT	66.19	3.47	50–72

*N = number; *SD* = standard deviation*.

### Results of Structural MRI Data

**Figure 2:**
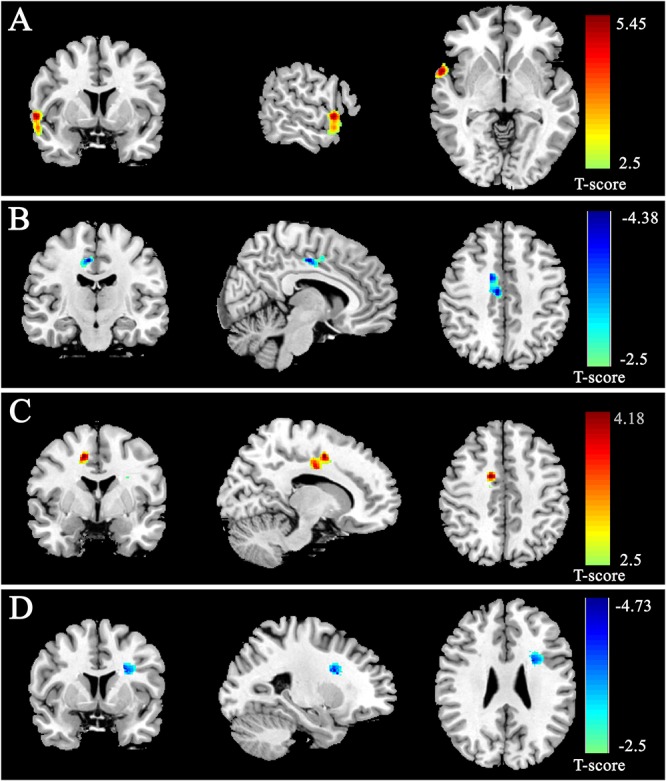
Gray matter density (GMD) and white matter density (WMD) were correlated with RAT scores. GMD was positively correlated with RAT scores in anterior STG **(A)** and negatively correlated with RAT scores in dorsal ACC **(B)**. WMD was positively correlated with RAT scores in dorsal ACC **(C)** and negatively correlated with RAT scores in IFG **(D)**. All results were shown at *t* > 2.5 for visualization purpose.

After controlling the effects of age, gender and the score of CRT, the multiple regression analysis showed that RAT was significantly positively correlated with the rGMD in the right STG and negatively correlated with the rGMD in the right dorsal anterior cingulate gyrus (dACC). Meanwhile, the analysis also revealed that RAT was significantly positively related with the rWMD in the right dACC and negatively related with the rWMD in the left inferior frontal gyrus (IFG) expended to pars opercularis. No other significant effects were found. The information of above brain regions was shown in Figure 2 and Table 2.

**Table 2:** Brain regions significantly correlated with the score of RAT.

Brain regions	H	MNI coordinates			Cluster size (mm^3^)	*t*-value (peak voxel)
		*x*	*y*	*z*		
**Gray matter density**						
Positive Correlation						
Superior Temporal Gyrus	R	60	6	-3	1474.9	5.45
Negative Correlation						
Anterior Cingulate Gyrus	R	7.5	-15	44.5	1454.6	4.38
**White matter density**						
Positive Correlation						
Anterior Cingulate Gyrus	R	13.5	-1.5	45	1339.9	4.18
Negative Correlation						
Inferior Frontal Gyrus	L	-25.5	6	27	1067.6	4.73

*H, hemisphere; L, left hemisphere; R, Right hemisphere; MNI, Montreal Neurological Institute. The *P*-value was set at *P* < 0.05 corrected by AlphaSim*.

## Discussion

In the present research, the anatomical basis of convergent thinking as measured by RAT was explored using VBM. As far as I knew, it was the first research to explore the relationship between individual convergent thinking measured by RAT and GMD/WMD. Results showed that the rGMD of the right STG was positively correlated with RAT, while the rGMD of the right dACC was negatively correlated with RAT. In addition, the results also revealed that the rWMD of the right dACC was positively correlated with RAT, while the rWMD of the left IFG was negatively correlated with RAT. These results corresponded to the findings that the STG was activated by remote association problem solving and ACC and IFG were involved in creative thinking and insightful problem solving (Jung-Beeman et al., 2004; Aziz-Zadeh et al., 2009; Dietrich and Kanso, 2010; Jung et al., 2010b).

Previous ERP studies had revealed that the STG was a common region involved in remote associates problems solving (Kounios et al., 2008; Qiu et al., 2008). Other research found that sentence and complex discourse increased the activation in STG which involved in semantic integration (Mazoyer et al., 1993; Stowe et al., 1999). Moreover, patients with right temporal damage would be in trouble during comprehending metaphors which emphasized distant semantic correlation (Brownell et al., 1990). Especially, Mark Jung-Beeman et al. (2004) used fMRI to explore the neuromechanism of RAT and found the increased activation in the right STG which supposed to conduct coarse semantic coding and accelerate the formation of remote associations. A recent review specialized in the function of the temporal lobe in insightful process and indicated that the aSTG was a critical hub in the novel association forming (Shen et al., 2017). Our findings further proved the notion that brain regions involved in some cognitive function would impact the efficiency and quality of the individual’s capacity to complete that function. Taken together, the positive association between the rGMD of the STG and the score of RAT might demonstrate that the right STG was particularly important for tasks which required the using of distant semantic associations between words.

The results also revealed that the rGMD of the right dACC was negatively associated with RAT and the WMD of the right dACC was positively correlated with RAT. Previous study suggested dACC involved in detecting conflicts (Botvinick et al., 1999; Enriquez-Geppert et al., 2013). It was suggested that ACC involved in the process of almost all types of creativity, such as insightful problem solving (Carlsson et al., 2000) and artistic creativity (Bengtsson et al., 2007; Berkowitz and Ansari, 2008; Kowatari et al., 2009). A recent research showed that the scores of creativity achievement questionnaire (CAQ) was positively associated with the rGMV in dACC and rostral ACC. Previous studies suggested that the ACC involved in the suppression of irrelevant thought, the shift of fixed mind-sets (Sawyer, 2011) and the development of general strategies in creative problem solving (Luo and Knoblich, 2007). Howard-Jones et al. (2005) discovered creative story generation activated the ACC which engaged in the selecting contextual information from episodic memory and monitoring extra conflict to form the novel and appropriate story. In consideration of the importance of dorsal ACC in different creative tasks, the critical node of executive network, these results might suggest that the executive network facilitated the process of creative performance.

Meanwhile, the results revealed that the rWMD of the left IFG which extended to pars opercularis was negatively associated with RAT. This was in accordance with research that proved that verbal creative task involved in IFG, supplementary area and premotor cortex (Brown et al., 2006; Mashal et al., 2007). Broca’s area, usually located in the triangular and opercular part of IFG in left hemisphere, was frequently engaged in verbal fluency (Costafreda et al., 2010) and semantic generation (Vidorreta et al., 2015). Other fMRI research also found the left IFG was involved in the generation of creative idea (Bechtereva et al., 2004), inventive conception (Zhang et al., 2014), matchstick problem task (Kleibeuker et al., 2013) and creative writing (Shah et al., 2013). R.E. Jung et al. (2010a) investigated the association between divergent thinking and white matter integrity measured by Fractional Anisotropy (FA) and found the score of divergent thinking task was negatively associated with the FA in the left IFG. In our study, the decreased rGMD in the left IFG was associated with convergent thinking. This might be because the cortical thinning was the inevitable process during cortical maturity. Sowell et al. (1999) found the gray matter density in the frontal lobe decreased from adolescence to adulthood. This reduction was thought be related to the increased efficacy of cognitive processing (Ernst and Korelitz, 2009). This was confirmed by the study’s finding that the decreased cortical thickness in frontal lobe was associated with intelligence in early childhood (Shaw et al., 2006) and the reduced cortical thickness in lingual gyrus was related with creative task (Shaw et al., 2006). Shen et al. (2018) also indicated that the left IFG was a part of the brain network involved in insight process and played an important role in the inhibitory of improper associations and the breaking of mental sets. In the present study, the decreased rGMD associated with higher RAT score might indicate that there was higher efficiency and quality of the left IFG in semantic generation and integration, which facilitated the performance of creative behavior.

In our findings, the score of RAT was associated with STG and IFG, and was partially consisted of the model of semantic processing named Bilateral Activation, Integration, and Selection (BAIS) (Jung-Beeman, 2005). This model supposed that semantic function consisted of three parts: semantic activation, semantic integration, and semantic selection. Three different brain regions lived in two brain hemispheres, posterior middle and superior temporal gyrus, anterior middle and superior temporal and inferior frontal gyrus, backed up these semantic parts, respectively (for more details M. Jung-Beeman, 2005). A great deal of research on creativity cognition indicated that one or more of the above brain regions were involved in creative performance (e.g., Jung-Beeman et al., 2004; Bengtsson et al., 2007; Abraham et al., 2012; Aziz-Zadeh et al., 2012; Jung et al., 2013; Kleibeuker et al., 2013; Beaty et al., 2014; Benedek et al., 2014; Zhang et al., 2014; Li et al., 2015). This model reinforced the relationship between creative cognition, semantic integration and semantic selection.

In the present research, the deceased and increased GMD were both found to be related with convergent thinking. But what larger or smaller was more better? Previous results suggested that neural plasticity might be expressed through reorganization of gray matter or white matter and reflected in the deceased and increased in disparate regions (Maguire et al., 2000; Draganski et al., 2006). Similar results also found the gray matter volume were positively and negatively correlated with creativity in disparate brain regions (Chen et al., 2014; Li et al., 2015). This question might be elucidated after the implementation of longitudinal or intervention in further studies. Our results further certified the notion that the functional information can be measured in white-matter and challenged the opinion that the blood oxygenation level-dependent (BOLD) signals in white matter was considered as noise (Logothetis and Wandell, 2004). Previous studies demonstrated the white matter BOLD signals, such as the low-frequency BOLD fluctuations (LFBFs) (Ji et al., 2017), resting-state functional connectivity (Jiang et al., 2018) and functional networks (Huang et al., 2018), can be reliably detected in the white-matter. These findings proposed that the WM signals may be of physiological significance. Other studies also found the relationship between WMD and creativity (Zhu et al., 2013; Fink et al., 2014; Chen et al., 2018).

## Conclusion

The present research used VBM to identify the GMD correlates of divergent thinking as measured by RAT. The results showed that a positive correlation between GMD in the right STG and RAT, while a negative correlation between GMD in the right dACC and RAT. In addition, the WMD in the dACC was positively correlated with the RAT. These results indicate that higher convergent thinking might be related to the enhanced ability of sentence comprehension, information integration and conflict monitoring. However, several limitations should be noticed. Because the young undergraduate participants with high-level education and right-handed subjects were enrolled, the external validity of our research would be affected. Mismatch of the sex distribution of our study was not matched perfectly (117 men versus 144 women). Although we found the relationship between convergent thinking and brain gray matter, we could not answer the question about the direction of relationship between convergent thinking and the increased/decreased rGMD. Future longitudinal or intervention investigations might promote the solution of the complex relationships between convergent thinking and brain structure. Moreover, because the present research adopted only the rGMD to explore the structural basis of RAT, more method could be used in the investigation of the neural mechanisms of RAT in future. Given that convergent thinking consisted of various task, such as anagram word puzzles (Kounios et al., 2008) and Chinese logogriphs (Qiu et al., 2010), the brain structural basis of other convergent thinking tasks could be explored in the future study.

## Author Contributions

WL designed the study, collected and analyzed the data, and wrote the paper. JQ and QZ provided the idea of the study, interpreted the results, and revised the paper. GL and BJ revised the paper.

## Conflict of Interest Statement

The authors declare that the research was conducted in the absence of any commercial or financial relationships that could be construed as a potential conflict of interest.

## Footnotes

1 http://fcon_1000.projects.nitrc.org/indi/retro/southwestuni_qiu_index.html

2 http://dbm.neuro.uni-jena.de/vbm/

3 https://www.fil.ion.ucl.ac.uk/spm/software/spm8/

4 http://restfmri.net/forum/REST_V1.8
